# Research Progress on Atomically Dispersed Fe-N-C Catalysts for the Oxygen Reduction Reaction

**DOI:** 10.3390/molecules29040771

**Published:** 2024-02-07

**Authors:** Yuebin Lian, Jinnan Xu, Wangkai Zhou, Yao Lin, Jirong Bai

**Affiliations:** 1 School of Optoelectronic Engineering, Changzhou Institute of Technology, Changzhou 213032, China; 2 School of Chemistry and Environmental Engineering, Jiangsu University of Technology, Changzhou 213001, China; jinnan_xu@126.com (J.X.);; 3 Research Center of Secondary Resources and Environment, School of Chemical Engineering and Materials, Changzhou Institute of Technology, Changzhou 213022, China; xiaoduovozz@163.com

**Keywords:** atomically dispersed, Fe-N-C, mechanism investigation, activity enhancement strategy, oxygen reduction reaction

## Abstract

The efficiency and performance of proton exchange membrane fuel cells (PEMFCs) are primarily influenced by ORR electrocatalysts. In recent years, atomically dispersed metal–nitrogen–carbon (M-N-C) catalysts have gained significant attention due to their high active center density, high atomic utilization, and high activity. These catalysts are now considered the preferred alternative to traditional noble metal electrocatalysts. The unique properties of M-N-C catalysts are anticipated to enhance the energy conversion efficiency and lower the manufacturing cost of the entire system, thereby facilitating the commercialization and widespread application of fuel cell technology. This article initially delves into the origin of performance and degradation mechanisms of Fe-N-C catalysts from both experimental and theoretical perspectives. Building on this foundation, the focus shifts to strategies aimed at enhancing the activity and durability of atomically dispersed Fe-N-C catalysts. These strategies encompass the use of bimetallic atoms, atomic clusters, heteroatoms (B, S, and P), and morphology regulation to optimize catalytic active sites. This article concludes by detailing the current challenges and future prospects of atomically dispersed Fe-N-C catalysts.

## 1. Introduction

Escalating energy demand and mounting environmental concerns have underscored the importance of developing clean energy conversion technologies. Among these, fuel cells (FCs) and metal–air batteries (MABs) are emerging as vital sustainable and clean energy conversion technologies [1,2,3,4,5]. However, the efficiency of these technologies is often hampered by the kinetics of the oxygen reduction reaction (ORR), a crucial component of energy conversion. This results in high overpotential, low energy efficiency, low power density, and poor cyclic stability of catalytic reactions. Currently, the most effective ORR catalysts are based on precious metals such as platinum (Pt) and its alloys. However, the rising cost of these materials, particularly in the context of proton exchange membrane fuel cells (PEMFCs), has posed significant challenges to their large-scale practical applications [6,7,8,9]. Despite the ability of Pt-based catalysts to optimize the performance of air electrodes in fuel cells, their prohibitive cost and dwindling resources have impeded the commercialization of zinc–air batteries (ZABs) and fuel cells. Therefore, the development of more stable, efficient, and cost-effective ORR electrocatalysts is pivotal for the widespread adoption of these energy conversion devices [10,11,12,13].

Among the various non-precious metal catalysts proposed as alternatives to precious metal catalysts, atomically dispersed M-N-C catalysts (including Fe, Mn, and Co) have demonstrated promising activity and stability for ORR. This is attributed to their unique electronic structure [3,14,15,16,17]. These atomically dispersed catalysts can maximize atom utilization, use metal resources rationally, and enhance the efficiency of the atom economy. The active centers of these catalysts are derived from their unsaturated coordination environment and distinct electronic structure. Furthermore, the superior atomic dispersion of the catalyst allows it to mimic the electronic interactions and spatial effects of the substrate, thereby boosting its catalytic performance. With their remarkable atomic utilization efficiency and exceptional electrocatalytic activity, atomically dispersed catalysts hold the potential to enhance the electrocatalytic kinetics of ORR [18,19,20].

M-N-C single-atom catalysts (SACs) need to be extremely small to achieve a high performance, making the enhancement and utilization of metal sites a primary task for improving ORR activity and stability. Atomically precise electrocatalysts, including single atom, diatomic, and clustered polyatomic electrocatalysts, have garnered considerable attention in recent years. Among these, iron-based atomically dispersed ORR electrocatalysts have been the subject of extensive research [21,22]. Experimental results and theoretical calculations have demonstrated that Fe-Nx-structured activated carbon catalysts outperform transition metal-based activated carbon catalysts such as Co, Ni, and Mn in terms of oxygen reduction activity. They exhibit excellent oxygen reduction performance with four-electron selectivity. As a result, atomically dispersed Fe-N-C, with its suitable adsorption/desorption capacity for oxygen-containing species in the catalytic process, is considered to be the most promising non-precious metal electrocatalyst to replace platinum-based materials [23,24,25]. Furthermore, the Fe-Nx structure not only exhibits a lower kinetic barrier due to the reduction in free energy from O* to OOH*, but the robust bonding between the Fe and N atoms also contributes to its superior catalytic stability.

Despite significant breakthroughs in the catalytic activity of Fe and N co-doped carbon-based ORR electrocatalysts, their direct application to the cathode of PEMFCs is still challenged by poor stability and serious durability issues. These issues are mainly due to the degradation of the catalyst layer, partial Fe deactivation, and carbon corrosion, which lead to the loss of active sites and proton transfer performance, thereby affecting the overall activity and stability. This hinders their practical application. Therefore, understanding and analyzing the degradation mechanism of carbon-based catalysts in PEMFCs and identifying effective strategies to improve their stability/durability are of great theoretical and practical value. This paper reviews the common strategies and methods for improving the stability and activity of Fe-N-C catalysts, based on the investigation of their activity origin and degradation mechanism, and discusses the existing challenges.

## 2. Mechanism Investigation

### 2.1. General ORR Mechanism

The intrinsic ORR activity of the Fe-N_4_ site is linked to the electronic structure of carbon supports. By controlling the electronic structure of these supports, the interaction between the supports and Fe-N_4_ site can be altered, leading to changes in the activity of the Fe-N_4_ site. However, the essence of this change in Fe-N4 ORR activity remains a mystery due to the complex factors influencing it. To comprehend the Fe-N_4_ ORR activity, numerous attempts have been made to identify the descriptors of Fe-N_4_ ORR activity. For instance, the full width at the half-maximum of C 1s photoemission spectra [26], d-band center [27], and the electronegativity of the nearest neighbor atoms [28] are considered to describe the ORR activity of the Fe-N_4_ site. However, these descriptors struggle to explain the orientation-dependence and differences in ORR activity at the same Fe-N_4_ site on different carbon supports. Liu and his team have proposed a model to understand the ORR activity of the Fe-N_4_ site, which is based on the spatial structure and energy level of the frontier orbitals, as determined by density functional theory calculations. Using the regulation of divacancy defects on the Fe-N_4_ site’s ORR activity as an example, they demonstrate that the hybridization between Fe 3dz^2^, 3dyz (3dxz) and O_2_ π* orbitals is the origin of Fe-N_4_ ORR activity. Their team discovered that descriptors such as the Fe-O bond length, the d-band center gap of spin states, the magnetic moment of the Fe site, and *O_2_ can accurately predict the ORR activity of the Fe-N_4_ site. Moreover, these descriptors and the ORR activity of the Fe-N_4_ site are primarily distributed in two regions, with significant differences that are strongly related to the height of the Fe 3d projected orbital in the Z direction [29].

### 2.2. Origin of Activity

Atomically dispersed Fe-N-C catalysts are among the most active non-precious-metal catalysts for the ORR. These catalysts are primarily synthesized through thermal decomposition. However, the process of converting Fe, N, and C precursors into Fe-N_x_ active sites during thermal decomposition is not fully understood. This gap in understanding complicates the establishment of an accurate relationship between synthesis conditions and active site structures. Consequently, the true catalytic active sites remain a topic of debate. This uncertainty has led to the reliance on trial and error and enumeration methods for improving activity and stability in experimental processes, significantly hindering the practical application of atomic Fe-N-C catalysts.

**Figure 1:**
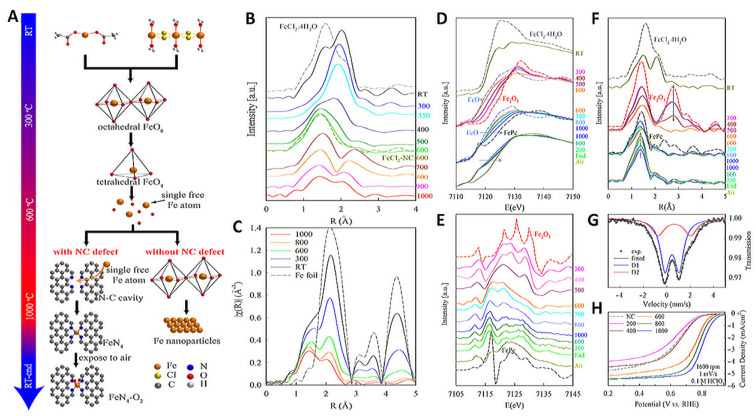
(**A**) Schematic illustration of the thermal evolution of iron compounds up to 600 °C. (**B**,**C**) Fourier transform extended X-ray absorption fine structure (FT-EXAFS) spectra. (**D**) X-ray absorption near edge structure (XANES). (**E**) Fourier transform (FT)-EXAFS. (**F**) The first derivative of the XANES spectra. (**G**) A 57Fe Mössbauer spectrum. (**H**) Rotating disk electrode (RDE) polarization plots of FeCl_2_-NC-T catalysts [31]. Copyright © 2023 American Chemical Society.

Research indicates that the adsorption of Fe^3+^ leads to the formation of FeO_x_ particles. These particles transform into atomically dispersed Fe-N_4_ coordination structures during thermal activation, which increases the density of Fe-N_4_ active centers. This transformation is primarily due to the higher thermal stability of Fe-N_4_ structures compared to FeO_x_. Various factors, including coordination number, symmetry, and changes in the length of the Fe-N bonds in the Fe-N_x_ coordination structure, can alter the charge distribution of the central Fe ion and the surrounding C atoms. These alterations influence the adsorption of O_2_ on the Fe-N_4_ site and the cleavage of O-O bonds, ultimately determining the performance of the catalyst [30]. Deborah J. Myers and her team conducted an in-depth study on the changes in the geometric and electronic configuration of the active center Fe atom during in situ high-temperature pyrolysis using X-ray absorption spectroscopy (Figure 1) [31]. Their findings shed light on the complex process of converting the precursors to the Fe-N_x_ active sites during high-temperature pyrolysis. The team discovered that the Fe precursor transforms into Fe oxide below 300 °C. Subsequently, a tetrahedral Fe_1_(II)-O_4_ structure forms through a crystal-melt transition below 600 °C. Importantly, above 600 °C, Fe_1_(II)-O_4_ releases a single Fe atom, leading to the formation of Fe_1_(II)-N_4_ active sites. Based on these insights, the authors suggest a novel non-contact pyrolysis method. In this method, the metal and the nitrogen-doped carbon (N-C) substrate do not have direct physical contact. At high temperatures, gaseous metal atoms are captured by nitrogen defects through gas-phase metal single-atom migration mechanisms, forming active sites.

**Figure 2:**
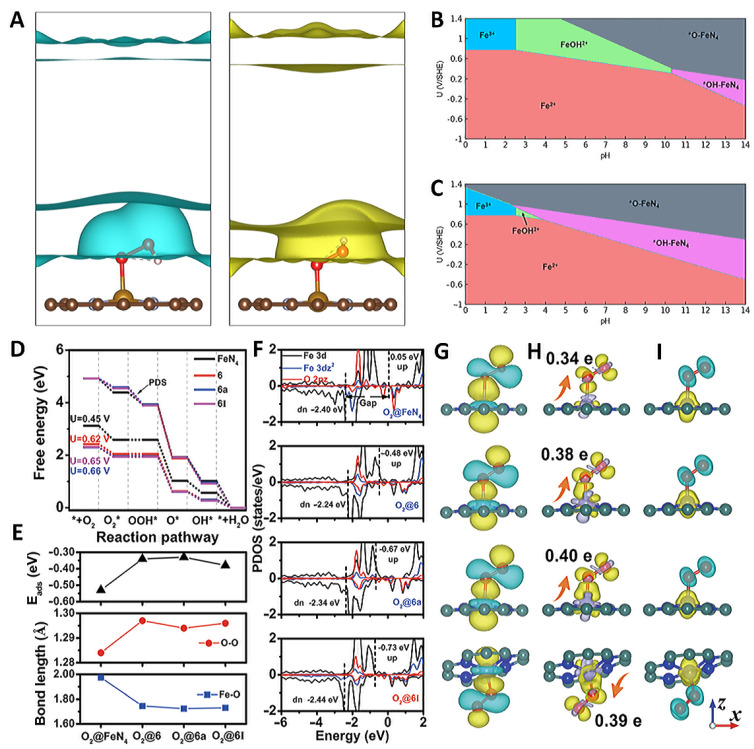
(**A**) Ionic countercharge density for *OOH corresponding to −2.01 V/SHE and 1.88 V/SHE. Stability diagram of the (**B**) pyridinic FeN_4_C slab and (**C**) pyrrolic FeN_4_C slab [32]. Copyright © 2023 American Chemical Society. (**D**) Gibbs free energy diagrams, (**E**) adsorption energy, (**F**) PDOS, (**G**) maximally localized Wannier functions of Fe 3d_z_^2^, O_2_ 2pz orbital, (**H**) charge density difference, (**I**) spin density [29]. Copyright © 2023 Springer Nature.

Zhou and his team conducted an extensive investigation of 13 different ortho-ligand FeN_x_C conformations and their corresponding ORR activities. They simulated a realistic electrocatalytic environment using a constant potential implicit solvent model (Figure 2A,B) [32]. The study clarified the mechanism of how different types of ligand nitrogens (pyridine and pyrrole nitrogen) and pH influence the ORR catalytic activity. The findings revealed that pyrrolic nitrogen enhances the ORR activity more effectively than the ligand pyridine nitrogen. Among the configurations studied, FeN_4_C containing pyrrolic nitrogen exhibited the highest activity in an acidic medium. Moreover, the high ORR performance of Fe-N-C in alkaline electrolytes was attributed to the in situ conversion of the active center to *O-FeN_4_C and *OH-FeN_4_C. Liu et al. investigated the energy levels of the frontier orbitals and the spatial structure to elucidate the origin of the activity of the Fe-N_4_ sites through density functional theory (DFT) calculations, and the origin and correlation of these descriptors were summarized (Figure 2D–I) [29]. The results show that the Fe-O bond length (L_Fe-O_) can accurately describe the ORR activity of the Fe-N_4_ site. From the electronic configuration point of view, the spin state of the magnetic moment of the Fe site, the d-band center gap, and *O_2_ are almost linearly related to the ORR activity of the Fe-N_4_ sites.

### 2.3. Degradation Mechanism

Atomically dispersed Fe-N-C catalysts, due to their efficient atomic utilization and remarkable catalytic activity, are considered to be the most promising alternatives to platinum group metal (PGM) electrocatalysts. However, optimizing the performance of Fe-N-C presents a significant challenge due to a trade-off between stability and activity. Their stability in PEMFCs is not satisfactory, leading to rapid performance degradation. Currently, the degradation mechanism of Fe-N-C in PEMFCs is not well understood, and the complex phenomena and difficulties in understanding the mechanisms of Fe-N-C cathode deactivation hinder the development of solutions to enhance stability. There is a lack of in-depth electrochemical in situ analysis. The degradation mechanism of M-N-C SACs primarily involves protonation of N active sites, oxidation of free radicals on the catalyst, and detachment of metal species from the active site. The main cause of performance degradation of Fe-N-C SACs in PEMFCs is Fe demetallation and carbon corrosion. Fe-N_x_ sites in Fe-N-C catalysts undergo metal detachment, resulting in the loss of active sites. Simultaneously, carbon substrates in the catalyst layer undergo corrosion, reducing the ORR rate around the active sites [33,34,35].

**Figure 3:**
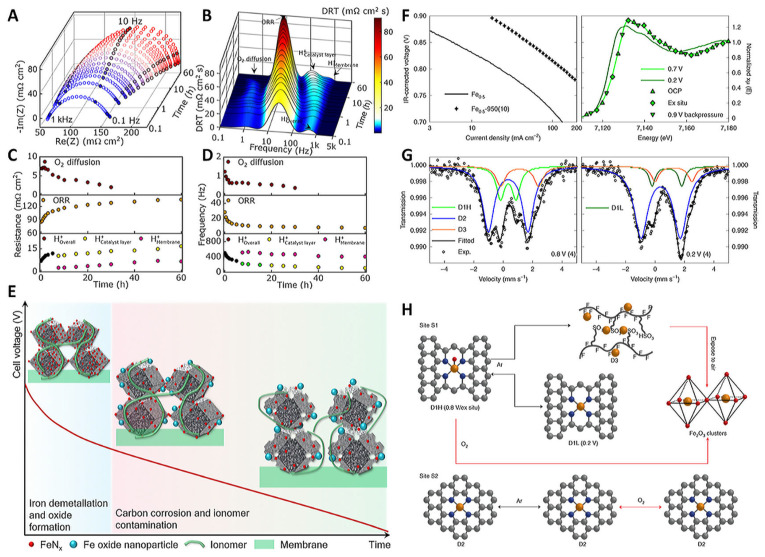
(**A**) Nyquist plot. (**B**) DRT plot. (**C**) Resistance and (**D**) characteristic frequencies. (**E**) Degradation mechanism of Fe-N-C at different reaction stages [36]. Copyright © 2023 The Royal Society of Chemistry. (**F**) Tafel plots and Fe K-edge XANES spectra, (**G**) in situ ^57^Fe Mössbauer spectra. (**H**) Structural changes [37]. Copyright © 2020 Springer Nature.

Zhao et al. employed advanced electrochemical analysis methods to investigate the rapid degradation phenomenon and unveiled the complexity of these degradation mechanisms through cyclic voltammetry and relaxation time distribution analysis (Figure 3A–E) [36]. After operating a practical proton exchange membrane fuel cell (PEMFC) with an Fe-N-C catalyst for 60 h, they discovered that up to 75% of the active sites in the catalyst layer were deactivated due to iron demetallation. The amount of carbon corrosion material in the catalyst layer increased fivefold, the proton transfer kinetics decreased fourfold, and the pathways for gas, ions, and electrons to the catalytic sites were extended, resulting in a threefold decrease in the ORR rate.

**Figure 4:**
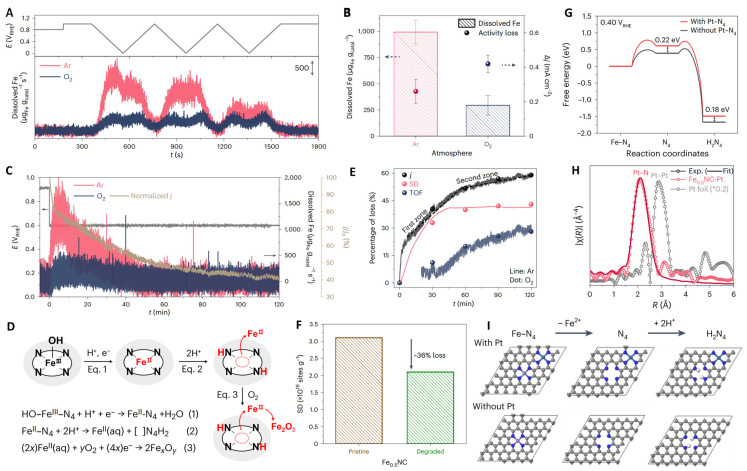
(**A**) Real-time Fe dissolution of Fe_0.5_NC/GDE. (**B**) ORR activity loss and Cumulative amounts of dissolved Fe. (**C**) Real-time Fe dissolution of Fe_0.5_NC/GDE (at 0.6 VRHE). (**D**) Proposed mechanism for Fe demetallation. (**E**) Relative loss of TOF, SD, and j values. (**F**) Site density value of pristine and degraded Fe_0.5_NC. (**G**) Free energy profiles of the Fe dissolution process. (**H**) Pt L3-edge FT-EXAFS spectra. (**I**) DFT-optimized structures [38]. Copyright © 2023 Springer Nature.

Frédéric Jaouen et al. conducted a study on Fe-N-C electrocatalysts with more stable Fe-N_x_ sites and fewer non-durable FeN_x_ sites using in situ spectroscopy, ex situ spectroscopy, and end-of-test spectrum analysis (Figure 3F,G) [37]. Their in situ 57Fe Mössbauer spectroscopy analysis revealed that Fe-N-C SACs contain two types of Fe-N_x_ sites, which have similar isomer shifts. Although these sites are common in Fe-N-C, their respective activities and lifetimes are not well understood. The researchers demonstrated that some S1 sites are stable using in situ 57Fe Mössbauer spectroscopy in an anoxic PEMFC, and they reversibly transform from high-spin iron to high-spin ferrous iron. Meanwhile, the electronic state of S2 is potential independent and is low- or intermediate-spin ferrous iron (Figure 3H). Chang Hyuck Choi et al. systematically investigated the changes in Fe-N-C active site density and conversion frequency over time under temperature/gas-controlled gas diffusion electrode conditions (Figure 4) [38]. Their results showed that the diagnosis of iron leaching identified a strong dependence of the in situ density changes on the operating parameters and plotted the stability over the lifetime. The changes in the main degradation mechanisms during operation were successfully revealed. The researchers also developed a proof-of-concept strategy using separated Pt ions as a non-catalytic stabilizer to improve fuel cell stability by reducing iron dissolution.

### 2.4. Resistance to Poisoning

In fuel cells, oxygen often unavoidably mixes with small organic molecules (SOMs). As a result, an important aspect of high-performance ORR catalysts is their excellent resistance to SOM poisoning. Many SOMs are easily adsorbed on the surface of Pt and oxidized, which reduces the ORR catalytic activity of the active site. Consequently, current commercial Pt/C catalysts often exhibit poor resistance to SOMs [39]. Fe-N-C catalysts are widely believed to have excellent resistance to SOM poisoning. However, a study by Sun’s group showed that methanol and ethanol significantly inhibited the ORR process of Fe-N-C catalysts rich in micropores (PDA-Fe-N-C) in an alkaline environment. The investigation of the size, polarity, and inhibition ability of organic molecules revealed that SOMs with a low polarity and larger size had a more pronounced inhibitory effect on the catalyst performance. The inhibition is believed to be due to the filling of the micropores by the SOMs, leading to a decrease in the mass transfer capacity. Interestingly, this suppression phenomenon did not occur in an acidic environment. This could be attributed to the protonation of the N group, which changes the polarity of the micropores and reduces the adsorption of SOMs [40].

## 3. Performance Improvement Strategy

### 3.1. Heterogeneous Atom Doping

Heteroatom doping can induce lattice distortions in electronic density, thereby activating catalytic active sites in the material. This typically introduces intermediate electronic states that act as a bridge to lower the charge transfer energy barrier between the catalyst and the surface adsorbate. Dopants can modify the electronic energy levels and local electronic distribution around them, making them active sites for catalytic reactions. Doping carbon with one or more active atoms can effectively optimize the bonding and antibonding states by adjusting the energy levels of the valence orbitals. The introduction of active atoms increases the energy of the active orbital and reduces the occupation of the antibonding state, which can optimize the adsorption of intermediates and improve the efficiency of the catalytic reaction. This fundamental physical principle is the basis for enhancing the catalytic performance of heteroatom-doped carbon. Theoretical studies have shown that the doping of heteroatoms is particularly attractive for improving ORR performance because they can drive defects into a confined state near the Fermi level [41,42,43,44,45,46,47,48,49,50,51,52,53,54,55,56,57]. The electronegativity and atomic size of P are lower than that of N, so doping P can optimize the spin density and local charge, while doping S in the graphene skeleton can modify the electronic configuration [41,49]. The co-doping of various non-metallic heteroatoms is an effective way to optimize the adsorption of oxygen-containing intermediates, regulate the local coordination environment, and improve the catalytic performance. In particular, co-doping with P can promote the transfer of electrons from C to N and the formation of more active sites, while the presence of S and N can break the uniformity and induce charge redistribution in the carbon skeleton [50,51,52]. Similarly, hybridization between the sp-orbitals of B and the d-orbitals of the transition metal contributes to the optimized properties of the d-band [53]. The coordination effect of heteroatoms will undoubtedly provide a new opportunity for the study of ORR in the future.

**Figure 5:**
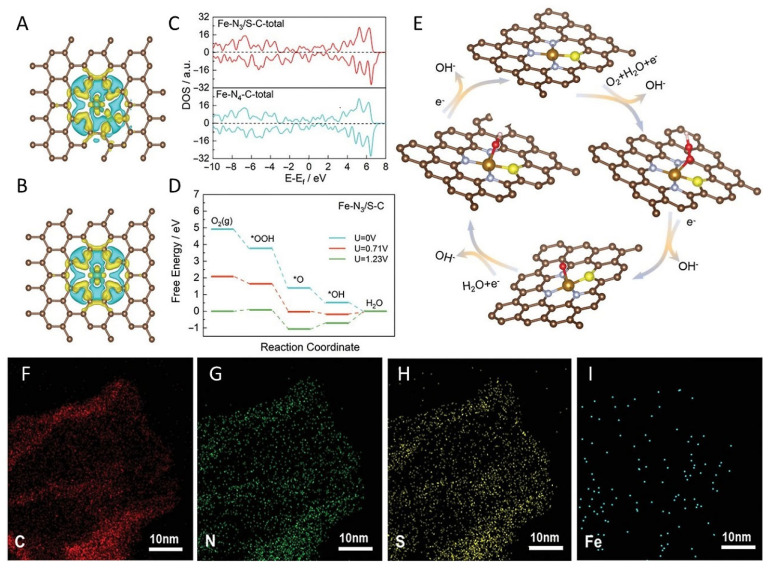
Charge density distribution of (**A**) Fe-N_3_/S-C and (**B**) Fe-N_4_-C. (**C**) DOS of Fe-N_3_/S-C and Fe-N_4_-C. (**D**) Free energy diagram, (**E**) reaction pathways of Fe-N_3_/S-C; Fe (brown), N (grey), S (yellow). (**F**–**I**) STEM image and elemental mapping; C (red), N (green), S (yellow), Fe (blue) [65]. Copyright © 2023 Wiley & Sons.

In addition, dopants can activate electron acceptor or electron donor states under different reaction conditions by creating favorable band gaps, electron structures, and state densities. This determines the number of free radicals and the energy consumption of charge transfer. Moreover, the derived active center significantly affects the interactions between reaction intermediates and the reduction in the electrode overpotential. The special configuration of some dopants can provide a durable reaction pathway. Breaking the symmetric electron distribution in the iron center of the single atom is an efficient way to optimize the intrinsic activity of the ORR. S [41,42,43,44,45,46,47,48], P [49,50,51,52,54,55,56,57,58,59,60,61,62,63,64], B [53], and other dopants provide an excellent method for enhancing the ORR performance of Fe-N-C. S atoms have a lower electronegativity and a larger atomic radius than N atoms. Therefore, Fe-N_3_S formed by S replacing N in Fe-N_4_ increases the outermost electrons of Fe atoms, resulting in a lower energy requirement for the ORR process compared to Fe-N_4_, effectively improving ORR performance. Tan et al. employed a straightforward template strategy and an advanced pyrolysis route to synthesize the Fe-N_3_S-C catalyst [65]. This catalyst exhibited an optimized pore structure and coordination environment, remarkably enhancing the active site density and catalytic performance (Figure 5). It also showed excellent ORR long-term durability and catalytic activity. The introduction of the S atom changes the electronic configuration of Fe-N_x_, which helps to improve its intrinsic catalytic activity. The layered mesoporous structure of the Fe-N_3_S-C catalyst is conducive to anchoring more iron atoms and exposing more active sites. Such a strategy could extend the application of traditional Fe-N-C electrocatalysts to more complex rechargeable ZABs.

**Figure 6:**
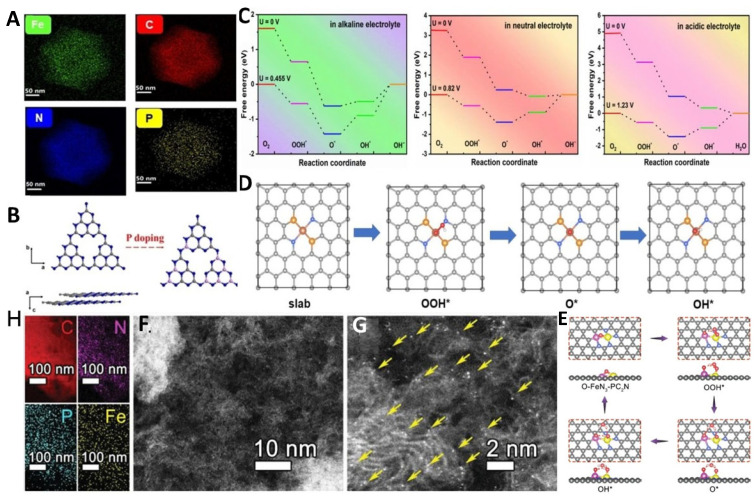
(**A**) Corresponding EDS results of Fe-SA/PNC. (**B**) Formation process of P-doped C_3_N_4_. (**C**) Gibbs free energy diagrams, (**D**) possible ORR process of Fe-N_2_P_2_; Fe (dark brown), N (blue), P (yellow) [57]. Copyright © 2023 Wiley & Sons (**E**) EDS elemental mapping images, (**F**,**G**) HAADF-STEM images of Fe,P-DAS@MPC; arrows (Fe SACs), (**H**) DFT-optimized adsorption configurations of FeN_3_–PC_2_N; C (red), N (purple), P (blue), Fe (yellow) [66]. Copyright © 2022 Wiley & Sons.

Compared to N and S doping, P doping in Fe-N-C SACs exhibits a stronger electron-donating ability. Similar to S doping, it can modify the electronic structure of Fe-N-C-based materials, reducing the adsorption energy of the ORR and thereby significantly enhancing the ORR activity. Xue et al. synthesized an atomically dispersed FeN_2_P_2_-based material, which demonstrated excellent ORR activity across the entire pH range. The half-wave potentials (E_1/2_) were 0.86 V for acidic electrolytes, 0.83 V for neutral electrolytes, and 0.92 V for alkaline electrolytes (Figure 6A–D) [58]. These are the best-performing catalysts reported to date. Moreover, there was no significant degradation in the performance after 30,000 cycles of durability testing, outperforming commercial Pt/C materials. FeN_2_P_2_ also exhibited excellent methanol tolerance. More importantly, the FeN_2_P_2_-assembled ZABs outperformed the Pt/C-assembled ZABs, indicating the potential of FeN_2_P_2_ for practical applications. Zong et al. reported an FeN_3_P-based material and proposed a strategy to construct iron and phosphorus dual-atom sites to enhance ORR activity and durability. The improved performance of the FeN_3_P-based material is attributed to the synergistic effect of the hydrogen bond interaction between the adsorbed and desorbed intermediates composed of neighboring Fe and P atoms (Figure 6E–H) [66]. On the other hand, since the P atom is not directly coordinated with the Fe atomic center, P doping in Fe-N-C helps regulate the electronic density of the Fe-N_4_ site, thereby enhancing its ORR performance. Simultaneously, this modified electrocatalyst also exhibits outstanding performance in ZABs and excellent cyclic stability.

### 3.2. Bimetallic Atom

Bimetallic atomic catalysts, composed of two different types of atoms, possess unique catalytic properties and activities. They exhibit a superior catalytic activity, stability, and multi-functionality compared to traditional SACs. By adjusting the interactions between atoms, these catalysts can achieve structural controllability. They are extensively used in ORR, water splitting for hydrogen production, and CO_2_ reduction, playing a significant role in energy conversion and environmental protection [67,68,69]. Through rational design and adjustment, the proportion, structure, and distribution of bimetallic atomic catalysts can be optimized for enhanced catalytic effects. As such, bimetallic atomic catalysts are considered to be the next generation of high-efficiency catalytic materials, with enormous potential in future catalytic research. However, further in-depth research is needed to fully exploit their advantages and overcome existing challenges.

**Figure 7:**
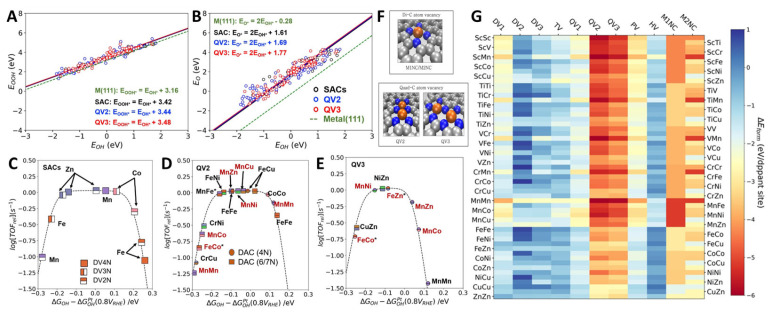
(**A**,**B**) Scaling relations of oxygen intermediates. (**C**–**E**) ORR activity volcano plots for the DACs and SACs. (**F**) Di-carbon atom vacancy site; Fe (golden), N (blue), C (grey). (**G**) Formation energy heat map for the DACs and SACs [77]. Copyright © 2023 Wiley & Sons.

As a unique type of catalytic material, bimetallic atomic catalysts have distinctive performance and application potential in catalytic reactions. With the increasing demands for catalyst activity and selectivity, the research and application of bimetallic atomic catalysts will continue to garner attention, playing a crucial role in energy conversion, environmental protection, and chemical synthesis. Bimetallic catalysts, when compared with monometallic catalysts, display synergistic effects. These effects are a result of metal–metal bonding interactions, which significantly enhance catalytic performance [70,71]. This improvement is demonstrated by DFT calculations, which show that the synergistic effects between bimetals can substantially reduce the reaction activation energy, which achieves the remarkable effect of 1 + 1 > 2 [72]. Bimetallic catalysts, due to their geometric (or strain) effect [73], electronic (or ligand) effect [74], and stabilizing effect [75], demonstrate excellent ORR activity, a high stability, and economic effectiveness [76]. Naiwrit Karmodak and colleagues identified single- and double-atom metal dopants from the first row of transition metals loaded on the surface of nitrogen-doped graphene containing defects for ORR using a catalyst calculation screening method (Figure 7) [77]. The study identified 4 SACs and 15 double-atom catalysts (DACs) with a high electrocatalytic performance based on the formation energy calculation and microscopic kinetic modeling of the reaction pathway. In the optimal SACs, Mn-N_x_ exhibits a high durability in both alkaline and acidic media. For the DACs, four potential candidates were identified, namely MnNi, CoCo, FeFe, and MnMn, all of which demonstrated excellent stability over a wide pH range.

**Figure 8:**
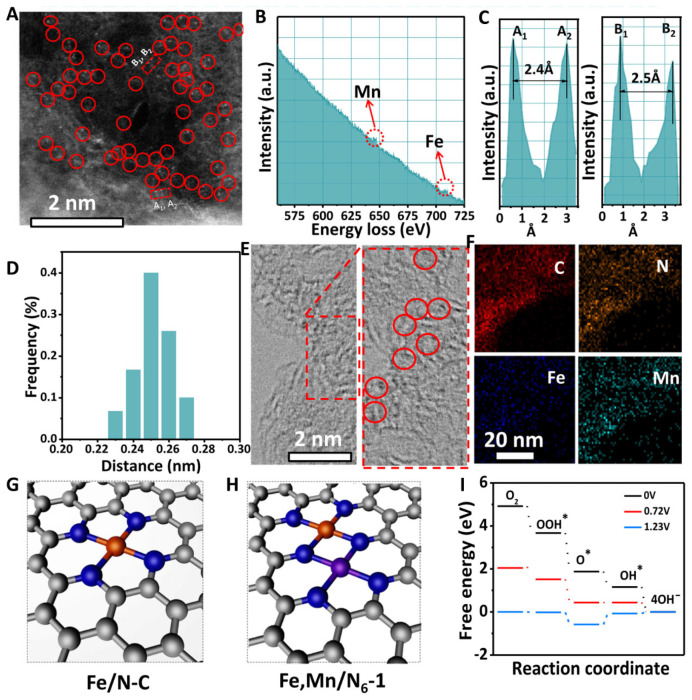
(**A**) HAADF-STEM image of bimetallic Fe/Mn sites, red circles (bimetallic Fe/Mn sites) (**B**,**C**) EELS of Fe-Mn sites. (**D**) Distance of Fe-Mn diatomic pairs. (**E**) HRTEM, (**F**) HAADF-STEM image and element mappings of Fe,Mn/N-C, red circles (bimetallic Fe/Mn sites). Optimized structures of (**G**) Fe/N-C and (**H**) Fe,Mn/N-C; Fe (orange) Mn (purple), N (blue), C (grey). (**I**) Free energy for Fe,Mn/N-C [105]. Copyright © 2021 Springer Nature.

In experimental research, a large number of diatomic electrocatalysts have been reported for the application of ORR. Among these, Fe-based diatomic catalysts mainly include FeMn [78,79,80,81,82,83,84,85,86,87,88], FeCo [89,90,91,92,93,94], FeCu [95,96], and FeNi [14,97,98,99,100,101,102,103,104]. Zhang et al. reported that doping of Mn atoms in Fe-N-C catalysts improves the ORR performance. The interaction between Mn and Fe is mainly due to the 3d orbitals, and the Mn-N structure in the Fe Mn-N-C catalyst can effectively activate the neighboring Fe^III^ sites through electronic regulation and the spin state transition (Figure 8) [105]. There is a significant overlap between Mn 3d and Fe 3d, allowing Mn^II^ to capture electrons from Fe^II^, resulting in a redistribution of electrons in Fe 3d. Moreover, the 3d orbital hybridization of Mn and Fe significantly reduces the energy band gap of Fe,Mn-N-C and numerous effective electron mass and increases the dispersion of the conduction band, thereby optimizing the electronic transport. The Fe,Mn-N-C electrocatalyst shows outstanding ORR performance due to the synergistic effects of Mn and Fe. In particular, it shows excellent durability in ZABs. Density functional theory (DFT) calculations further clarify the reason for the high ORR activity of Fe,Mn-N-C. When oxygen is adsorbed by the Fe-Mn bimetallic atom pair, it creates a suitable bond length, thereby reducing the dissociation energy barrier. Moreover, it can effectively trap oxygen-containing species and rapidly break the bond of M-OH, ensuring the regeneration of OH* and O*. In addition, the enhanced performance of ORR is also attributed to the effective inhibition of peroxide formation.

**Figure 9:**
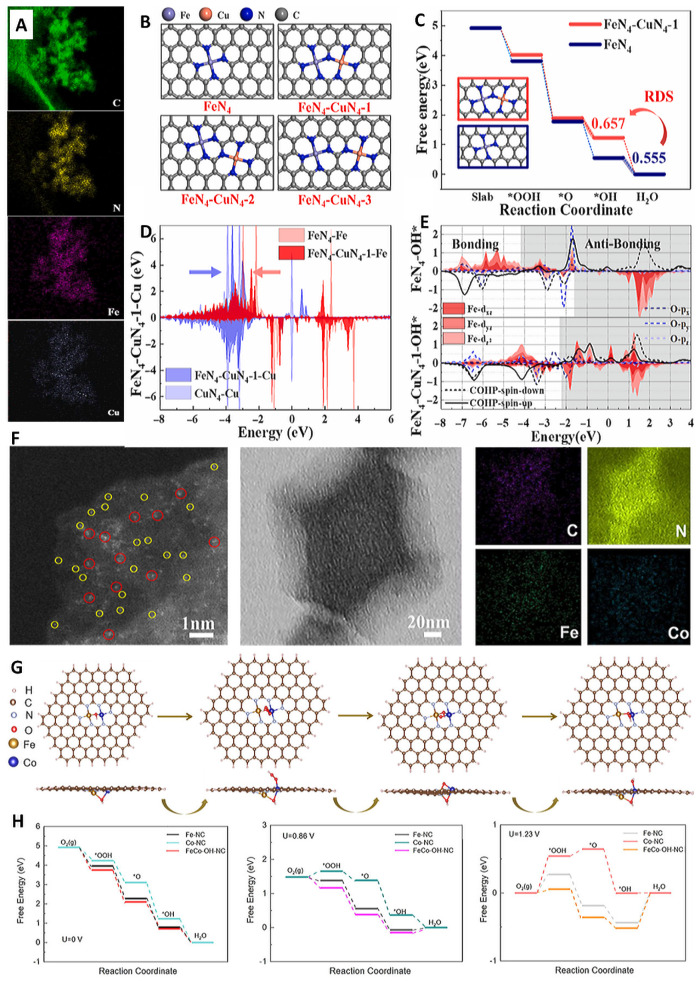
(**A**) Element mappings, (**B**) configurations diagram, and (**C**) free energy diagrams of FeN_4_-CuN_4_-1. (**D**) The d-band changes of Fe and Cu. (**E**) PDOS of the Fe and O orbitals [95]. Copyright © 2022, Elsevier Group. (**F**) AC-STEM image and elemental mapping; diatoms (red cycles), single atom (yellow cycles) (**G**) Side view and top view of the initial structures after adsorption. (**H**) Free energy for the ORR at a different potential [106]. Copyright © 2022, American Chemical Society.

In the volcanic diagram of ORR, Cu, as a non-noble metal, shows a better ORR performance compared to platinum. A significant amount of research has shown that the ORR activity of FeCu-N-C catalysts is superior to that of Fe-N-C SACs in various coordination structures. This is mainly due to the introduction of Cu, which can effectively change the electronic configuration of Fe SACs. Therefore, doping Cu metal atoms in Fe-N-C SACs is an efficient method to optimize their ORR performance. Xiao et al. reported FeCu-N-C DACs containing dual active sites of Fe-N_4_ and Cu-N_4_. Theoretical calculations were used to determine the Gibbs free energy of the sample’s catalyzing ORR [95]. It was found that the rate-determining step (RDS) in the presence of FeN_4_ alone is OH → H_2_O, as the strong adsorption of FeN_4_ on OH hinders the final electron transfer (Figure 9A–E). However, after introducing Cu-N_4_, the strain effect caused by the adjacent carbon environment of Cu-N_4_ replacing Fe-N_4_ attenuated the adsorption ability of the FeN_4_-CuN_4_ structure on OH, which leads to the RDS of FeCu-NC becoming O → OH, thereby enhancing the catalytic performance and kinetic process. He et al. reported a FeCo bimetallic electrocatalyst which exhibited excellent bifunctional catalytic activity. In addition, its excellent durability during ORR and oxygen evolution reaction (OER) processes was comparable to that of noble metal catalysts (Figure 9F–H) [106]. Calculations showed that the FeCo-N_6_ species can serve as the main active sites for both OER and ORR. The performance of FeCo-N-C was improved when compared to conventional single Fe and Co SACs. Moreover, the use of FeCo-N-C DACs for air cathodes in rechargeable and flexible ZABs resulted in high power density and long-term cycling durability.

### 3.3. Nanocluster Collaboration

Due to their adjustable electronic structure and excellent atomic utilization efficiency, SACs have attracted much attention [88]. However, there is still a need for continuous improvement in their single-atom activity and stability. Recent studies have shown that doping Fe-N-C materials with metal clusters can also improve their performance. In ORR catalysis research, metal nanoclusters can synergistically promote the activity of Fe-NC catalysts by adjusting their electronic structure and providing highly dispersed active sites, reducing the reaction energy barriers, improving the mass transfer performance of Fe-NC catalysts, and increasing the diffusion efficiency of reactants, thereby significantly enhancing the activity and improving the stability. At the same time, nanoclusters (NCs) can stabilize the active sites of Fe-NC catalysts, provide a protective layer to reduce oxidation and corrosion of the catalyst during the reaction process, and prevent aggregation and deactivation under reaction conditions. For example, some NCs can promote the electronic transfer of Fe atoms, thereby enhancing their reduction ability. Commonly studied metal clusters include Fe [107,108,109,110,111,112,113], Cu [114,115,116], Pd [117,118,119], Co [120,121], Ni [122,123], etc.

**Figure 10:**
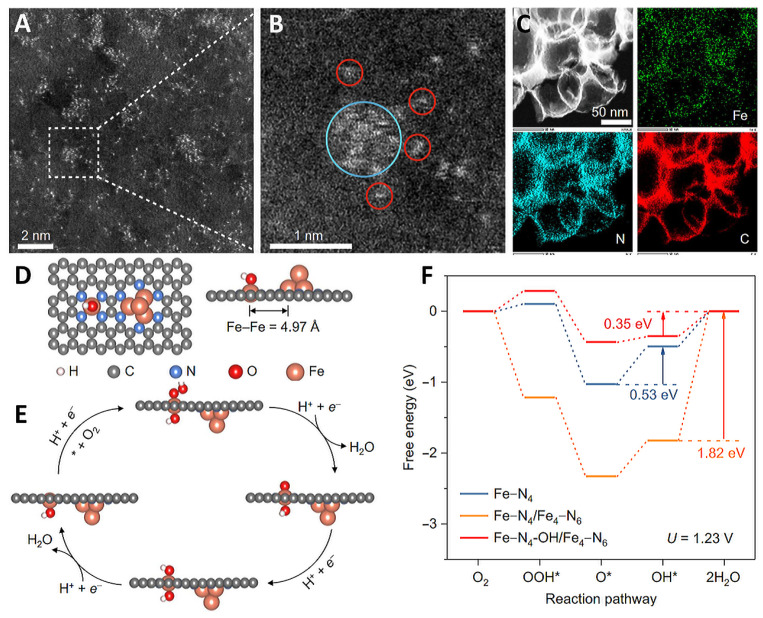
(**A**–**C**) HAADF-STEM image and corresponding element mappings; iron cluster (cyan circle) and iron atoms (red circles). (**D**) Model structure, (**E**) schematic ORR process. (**F**) Free energy diagrams of Fe-N_4_, Fe-N_4_/Fe_4_-N_6_, and Fe-N_4_-OH/Fe_4_-N_6_ [124]. Copyright ©2022 Springer Nature.

Shui et al. reported a novel Fe-based electrocatalyst that simultaneously possesses N-coordinated Fe NCs and closely spaced Fe-N_4_ active sites for acidic ORRs (Figure 10) [124]. In the thermal treatment process, N doping in carbonaceous support was used to achieve moderate coordination strength with metal elements while introducing Fe clusters, resulting in a uniform distribution of Fe monatomic and Fe NCs. The results show that there is a strong electron interaction between Fe NCs and Fe single atoms, and that Fe clusters can introduce OH ligands, reduce the ORR energy barrier and improve the performance of the Fe-N_4_ site. In addition, the stability of Fe-N_x_ species at different temperatures was predicted. The study found that Fe clusters optimized the adsorption strength of oxygen-containing species on the Fe-N_4_ site and shortened the amplitude of the Fe-N bonds of Fe-N_4_ through non-coherent vibrations of Fe clusters and single atoms. Therefore, metal dissolution on Fe-N_4_ sites was effectively suppressed, resulting in a 60% reduction in iron ion dissolution.

**Figure 11:**
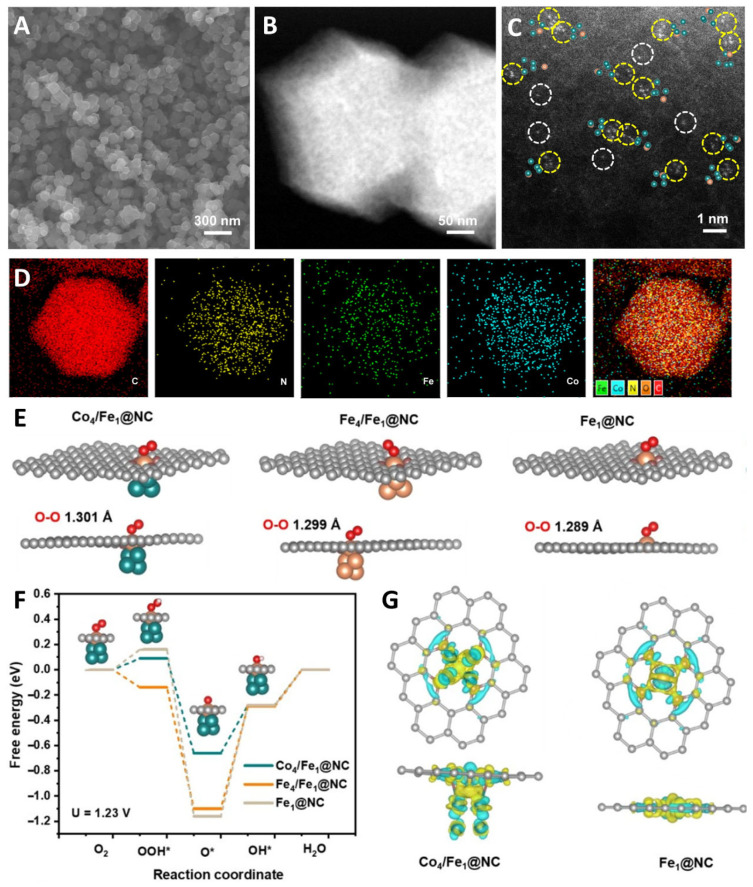
(**A**) SEM image, (**B**) aberration-corrected STEM image, (**C**) atomic-resolution HAADF-STEM image; C (red), N (yellow), Fe (green), Co (blue), (**D**) elemental mapping of Co_4_/Fe_1_@NC; Mechanism investigation of Co_4_/Fe_1_@NC. (**E**) O_2_ adsorption models of Co_4_/Fe_1_@NC, Fe_4_/Fe_1_@NC and Fe_1_@NC; (**F**) Gibbs free energy diagram for ORR on Co_4_/Fe_1_@NC, Fe_4_/Fe_1_@NC and Fe_1_@NC, (**G**) Charge density of Co_4_/Fe_1_@NC and Fe_1_@NC [125]. Copyright © 2023 Wiley & Sons.

**Figure 12:**
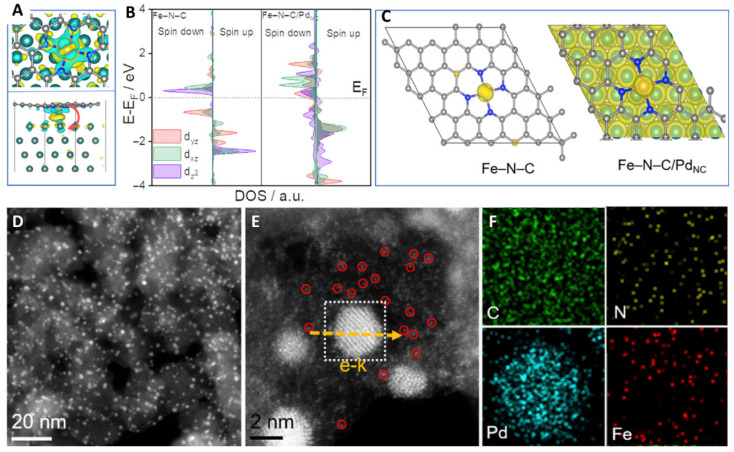
(**A**) Charge density difference, (**B**) DOS, (**C**) spin density of of Fe-N-C/Pd_NC_. (**D**,**E**) HAADF-STEM image; Fe single atoms (red circles), Pd_NC_ (white box),mapping area (yellow arrows) (**F**) EDS elementary of Fe-N-C/Pd_NC_ [126]. Copyright ©2023 Cell Press.

Han et al. reported a novel catalyst with Fe-N_4_ and Co_4_ clusters on a nitrogen-doped carbon support by encapsulating the clusters in a carbonaceous MOF precursor (Figure 11) [125]. This catalyst regulated the electronic structure of Fe-N_4_ through the Co_4_ clusters, resulting in a high stability and ORR performance. DFT theoretical calculations showed that neighboring Co_4_ clusters help to activate O_2_ at the Fe-N_4_ active site and reduce the adsorption of oxygen intermediates on Fe-N_4_, thereby reducing the overpotential of Fe-N_4_ during ORR and accelerating the overall ORR kinetics. Further charge density and d-band center theory results showed that the coupled Co_4_ clusters significantly optimized the electronic configuration of Fe-N_4_ and controlled its adsorption of oxygen-containing species. Wei et al. reported on the preparation of a spin-state-tunable Fe single-atom catalyst (Figure 12) [126]. In terms of non-precious metal loading, the stability and activity of Fe/Pd-N-C are better than commercial Pt/C catalysts in acidic electrolytes, mainly due to the electronic structure differences between Pd NCs and Fe single atoms. This allows Pd NCs to successfully induce an electron transition from the d_yz_ orbital to the d_z_^2^ orbital in the Fe single atom, achieving the transition of Fe(II) from low to medium spin. Further theoretical calculations show that the medium spin is favorable to the side-pair adsorption mode of O_2_, promoting the direct 4e^−^ pathway. At the same time, the occurrence of the 4e-dissociation pathway avoids the generation of H_2_O_2_, not only improving selectivity but also avoiding the attack of H_2_O_2_ on the active sites of Fe, carbon carriers, and proton exchange membranes, improving stability. In addition, the half-filled d_z_^2^ orbitals of the medium spin Fe(II) and the O 2p orbitals of OH* generate σ* bonds that facilitate the rapid desorption of OH*, effectively accelerating the entire ORR dynamics.

### 3.4. Active Site Density Engineering

The activity of the catalyst is closely related to the properties of the catalyst material (such as nitrogen content and the dispersion of metal species) and the microstructure of the catalyst (such as porosity and packing structure). In general, the higher porosity of the catalyst means that more active sites can be exposed to ensure rapid electron transfer. High-nitrogen isomers also tend to provide more active sites, while increasing the density of highly dispersed metal monomers can also significantly improve the ORR activity. Therefore, the use of the active site is not only dependent on the type of catalyst material but is also strongly influenced by the final microstructure of the electrocatalysts and the density of the active site. Disordered stacked structures tend to restrict the mass transfer channels and reduce the exposure of the active sites. The abundant porous structure is conducive to full exposure of the active site and promotes easier proximity of gas molecules and oxygenated intermediates to the active site during the reaction [127].

**Figure 13:**
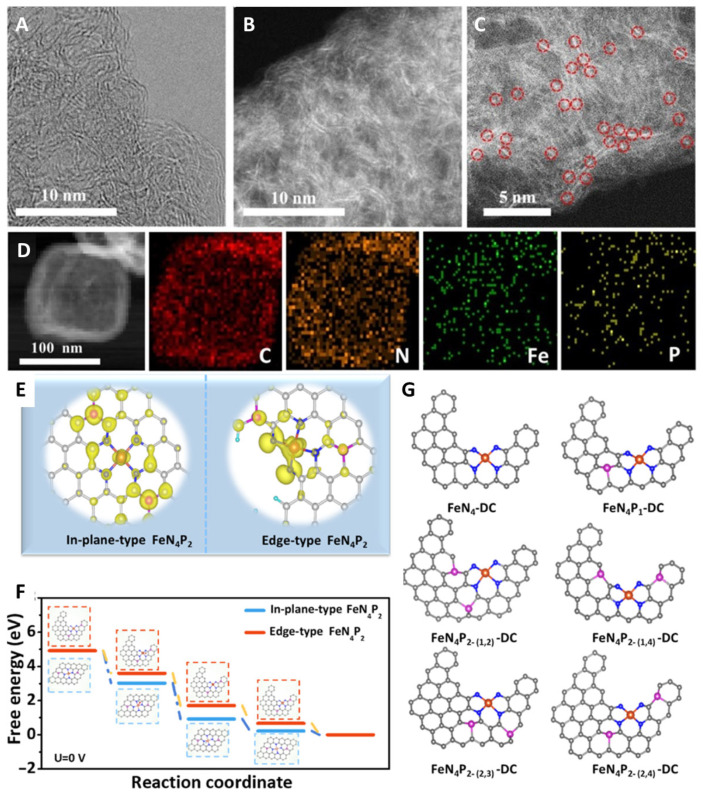
(**A**) HAADF-STEM images. (**B**–**D**) HRTEM image and the corresponding EDS elemental maps of Fe-N-C-P/N and P-C; circles (Fe-N-C-P SACs). (**E**) Differential charge densities, (**F**) free energy diagrams of in-plane-type and edge-type FeN_4_P_2_. (**G**) DFT models used for the stability calculations; Fe atoms (yellow), N atoms (blue), P atoms (carmine) [62]. Copyright © 2021 American Chemical Society.

To further improve the atomic utilization efficiency, Fe-N-C-based SACs can increase their ORR performance through active site density engineering [128,129], such as enhancing edge active sites [62,130], regulating structures [131,132,133,134,135,136,137,138,139,140,141], increasing loading [142,143], and other means to facilitate their performance. Yin et al. proposed a phosphorus-driven method to manipulate the electronic structure of edge FeN_4_ active sites to enhance ORR performance. This report demonstrated for the first time that local electron redistribution makes a significant difference between edge-type FeN_4_P_2_ and in-plane FeN_4_P_2_ of different charge densities, and that defects in the FeN_4_P_2_ structure favor the selectivity of the four-electron ORR pathway (Figure 13) [62]. After the introduction of P atoms onto a carbon-based substrate, the charge density around FeN_4_ undergoes a rearrangement and enhances the internal electronic conduction, thereby regulating the adsorption behavior of key intermediates. In particular, such delocalization can potentially cause atoms to be further apart, leading to significant interactions. The adjacent phosphorus active sites, which enhance activity, can drive significant long-range electronic delocalization of the Fe 3d center.

**Figure 14:**
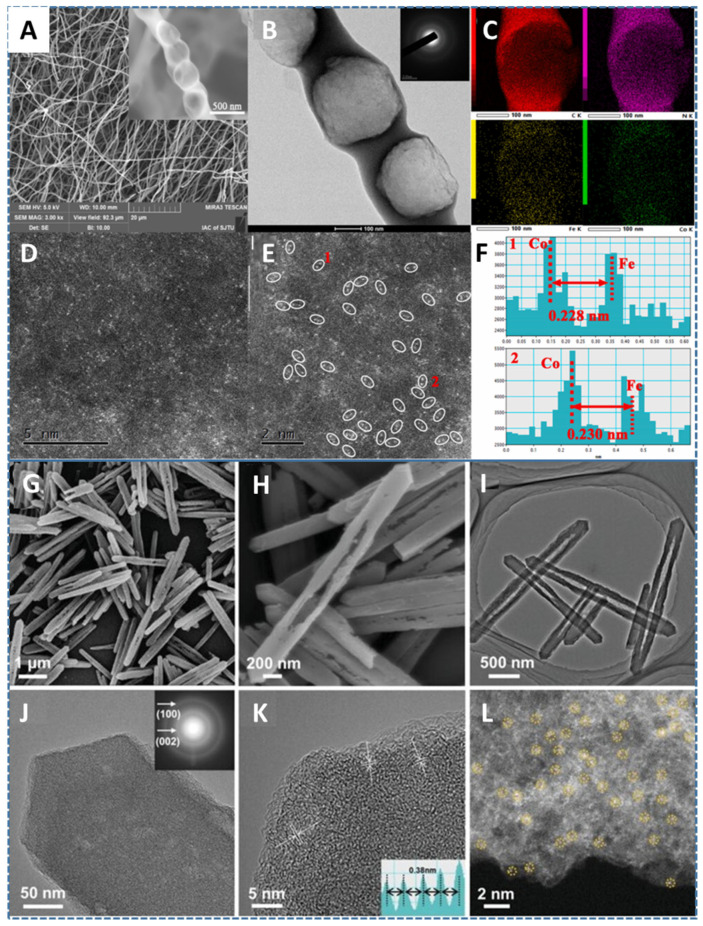
(**A**) SEM image, (**B**) TEM image, (**C**) EDS images, (**D**,**E**) HAADF-STEM images of Fe, Co SAs-PNCF, (**F**) distance of Fe, Co atom pair [143]. Copyright © 2022 Elsevier Inc. (**G**,**H**) SEM images, (**I**,**J**) TEM images, (**K**) HRTEM image, (**L**) HAADF-STEM image of Fe1-N-C HNRs; isolated Fe atoms (yellow dotted circles) [144]. Copyright © 2022, John Wiley & Sons.

Due to their unique electronic structure, SACs can effectively adsorb O_2_ and catalyze the subsequent O-O bond cleavage. However, SACs still suffer from low metal loading, so increasing the metal loading on Fe-N-C-based catalysts can also improve their ORR performance. Jiang et al. reported a method for preparing catalysts with up to 9.8 wt.% metal loading of dense FeCo DACs on porous nitrogen-doped carbon nanofibers using plasma defect engineering (Figure 14A–F) [143]. This method generates a large number of defects in the N-doped carbon nanofiber, providing the opportunity for high metal loading. Benefiting from the synergistic effects of Fe Co dual metal atomic sites, the FeCo-N-C catalyst showed outstanding ORR performance. In addition, a series of characterizations have revealed the dynamic structure and valence state changes of the active sites. This report not only provides a new strategy for the fabrication of highly loaded Fe-N-C SACs but also provides a mechanism study of dual-metal SACs in ORRs.

Ma et al. synthesized an open-cavity ZIF-8 hollow nanostructure and transformed it into a nitrogen-doped carbon hollow nanostructure to load Fe-N_4_ atomic active sites (Figure 14G–L) [144]. Compared with traditional ZIF-8-derived carbon, one-dimensional carbon nanomaterials have unique advantages due to their unique one-dimensional hollow structure, including efficient electronic transmission channels, a fast mass transfer, and a larger surface area. In addition, the high density of pyridine and graphite nitrogen doping can effectively regulate the microenvironment of the separated iron atoms, thereby reducing the activation energy barrier of the ORR process. These are effective methods for improving the ORR performance of Fe-N-C SACs. Notably, this catalyst also exhibits excellent performance in ZABs.

In addition, optimizing the degree of graphitization of carbon carriers is an effective strategy to potentially improve the oxidation resistance of carbon materials. The high degree of graphitization of the carbon carriers can further optimize the performance of the carbon materials. Highly graphitized carbon materials have a higher electrical conductivity, which helps to promote electron transfer in the reaction and can meet the requirements of various applications. Secondly, increasing the degree of graphitization of carbon materials can help to improve the oxidation resistance of catalysts, which can prolong their lifetime and reduce the performance degradation caused by oxidation [145,146,147]. Kang et al. reported an effective strategy (pyrolysis in high-temperature conditions (1100–1200 °C)) to significantly improve the stability of Fe-N-C catalysts. Pyrolysis in this temperature range allowed for the removal of the low-activity nitrogen atom uncoordinated sites, which are prone to produce harmful H_2_O_2_ side reaction products, and at the same time, it was possible to convert the less stable D1 site (O-FeN_4_C_12_) to the more stable D2 structural site (FeN_4_C_10_) in this temperature range [148]. Table 1 summarizes the performance of some recent atomically dispersed Fe-based ORR catalysts in different electrolytic media.

## 4. Conclusions and Outlook

Efficient and stable electrocatalysts play a crucial role in energy conversion. This article reviews the active sources and degradation mechanisms of atomic Fe-N-C SACs, as well as common modification strategies. Although significant progress has been made in ORR performance, the stability of M-N-C electrocatalysts still lags behind that of Pt/C. It is worth noting that the aim of M-N-C ORR electrocatalyst research should be to improve the catalyst to reduce the mass transfer loss under a high current density, and to further develop efficient hybrid/composite catalysts to improve their activity and durability in order to achieve successful application in PEMFCs. The key to replacing PGM catalysts with Fe-N-C catalysts lies in their atomic dispersion, abundant active centers, and high mass transport efficiency.

Future research must also focus on the following points:

(1) The precise design of the doping sites and the uniform distribution of the separated metal atoms require further research. There is an urgent need to increase the loading of individual metal atoms so that more individual atoms can be anchored to the substrate, resulting in greater activity and durability.
(2) Further research on different substrates in needed for the selection of an ideal, low-cost, stable support to improve the active site exposure and environmental stability of the catalyst. High-surface-area and high-volume porous carbon substrates are excellent substrates for active sites. A good substrate should be able to precisely regulate the physical/chemical environment to provide stronger bonds for isolated iron atoms, thereby maintaining stronger catalytic activity in the electrocatalytic process and ensuring long-term performance during the electrocatalytic process.
(3) In the design and preparation of catalysts, the framework structure and interatomic interactions of the support should be fully considered in order to maximize the dispersion of metal atoms, effectively suppress the aggregation of metal atoms, and reduce the loss of active sites. The scope of research on dopants should be further extended, and the effects of these impurities at different doping sites should be fully and accurately considered.
(4) Theoretical calculations and in situ characterization techniques should be used to further explore the relationship between structure and performance at the atomic level and to promote research into catalytic mechanisms. Advanced systematic testing methods are key factors in the rational evaluation of catalyst performance. With precise control of the coordination environment, Fe-N-C catalysts have broad application prospects in areas such as fuel cells (FCs).

In conclusion, the development of novel modified Fe-N-C catalysts for ORR is crucial, but in-depth research on catalyst synthesis, active sites, and reaction pathways is needed. In addition, theoretical simulations and experimental research should be systematically combined to elucidate the core control principles and reaction mechanisms of Fe-N-C catalysts and to reveal the changes in catalytic selectivity during catalyst design and optimization. The in-depth exploration of the relationship between the structure of Fe-N-C SACs and catalytic theory will provide insights into the design and optimization of energy conversion catalysts and help us to achieve green energy storage and conversion.

## Figures and Tables

**Table 1:** Comparison of the ORR performance of reported Fe-based catalysts.

Catalysts	Electrolyte	Half-Wave (V vs. RHE)	Current Intensity mA cm^−2^	Reference
Fe_2_-NC	0.1 M HClO_4_	0.78	5.5	[149]
Fe_SA_/Fe_AC_-NC 900	0.1 M HClO_4_	0.80	5.5	[107]
FeNi-N6	0.1 M HClO_4_	0.79	5.2	[150]
M/FeCo-SAs-N-C	0.1 M HClO_4_	0.851	4.7	[151]
Co_2_/Fe-N@CHC	0.1 M HClO_4_	0.812	5.9	[152]
FeN_4_Cl SAC	0.1 M HClO_4_	0.818	6.0	[153]
Fe-N/S-C-10%	0.5 M H_2_SO_4_	0.81	4.9	[154]
Fe-N-S CNN	0.5 M H_2_SO_4_	0.78	6.0	[155]
Fe_NCs_/Fe_SAs_-NC-Z8@34	0.1 M KOH	0.918	5.6	[108]
FeS/FeNSC	0.1 M KOH	0.91	5.3	[156]
Fe,P-DAS@MPC	0.1 M KOH	0.92	6.5	[66]
Fe@Fe/N-G-80	0.1 M KOH	0.866	6.34	[157]
Fe_ACs_/NPS HC	0.1 M KOH	0.87	6.0	[158]

## Data Availability

Not applicable.
